# Alzheimer-related individual factors modulate effects of transcranial direct current stimulation strength on white matter integrity in mild cognitive impairment

**DOI:** 10.1038/s41598-025-27612-7

**Published:** 2025-12-17

**Authors:** Jung-Won Lee, Sunghwan Kim, Suhyung Kim, Yoo Hyun Um, Sheng-Min Wang, TaeYeong Kim, Donghyeon Kim, Hyun Kook Lim, Chang Uk Lee, Dong Woo Kang

**Affiliations:** 1 Department of Psychiatry, College of Medicine, Seoul St. Mary’s Hospital, The Catholic University of Korea, 222, Banpo-Daero, Seocho-Gu, Seoul, 06591 Republic of Korea; 2 Department of Psychiatry, Yeouido St. Mary’s Hospital, College of Medicine, The Catholic University of Korea, Seoul, Republic of Korea; 3 Department of Psychiatry, St. Vincent’s Hospital, College of Medicine, The Catholic University of Korea, Seoul, Republic of Korea; 4 Research Institute, Neurophet Inc., Seoul, 06247 Republic of Korea

**Keywords:** Transcranial direct current stimulation, Mild cognitive impairment, White matter, Diffusion tensor imaging, Alzheimer’s disease, Prognostic markers, Dementia, Dementia

## Abstract

Transcranial direct current stimulation (tDCS) is a promising non-invasive intervention for mild cognitive impairment (MCI). This prospective study investigated the relationship between optimized electrical field (EF) strength of tDCS and white matter (WM) microstructural changes in 55 individuals with MCI. Magnetic resonance imaging (MRI)-based computational modeling was used to optimize EF strength targeting the left dorsolateral prefrontal cortex (DLPFC). Diffusion tensor imaging (DTI) assessed WM integrity through fractional anisotropy (FA), mean diffusivity (MD), and radial diffusivity (RD). Higher EF strength was significantly associated with increased FA and reduced MD and RD in specific left-lateralized tracts, including the anterior thalamic radiation, corticospinal tract, inferior fronto-occipital fasciculus, and inferior longitudinal fasciculus. These EF-dependent WM changes were moderated by Alzheimer’s disease (AD)-related factors. Greater WM plasticity was observed in Aβ-positive individuals, *APOE* ε4 non-carriers, and *BDNF* Met non-carriers. Moreover, *APOE* ε4 status significantly moderated the relationship between EF strength and executive function; in non-carriers, stronger EF strength was associated with improved Stroop performance, potentially reflecting enhanced WM integrity in the right superior longitudinal fasciculus. However, no significant associations were observed between EF-sensitive tracts and short-term cognitive changes in the full sample, suggesting that structural modifications may precede functional improvements or require longer follow-up. These findings emphasize the importance of individual AD-related factors in shaping neuromodulatory responses. They also support the need for longitudinal, sham-controlled studies to clarify the clinical implications of EF strength in personalized tDCS for MCI.

**Supplementary Information:**

The online version contains supplementary material available at 10.1038/s41598-025-27612-7.

## Introduction

Alzheimer’s disease (AD) is the most prevalent cause of dementia, characterized by amyloid-beta (Aβ) and tau protein accumulation, leading to progressive cognitive decline and functional impairment^1^. Mild cognitive impairment (MCI) is considered a prodromal stage of AD, distinguished by preserved independence despite measurable cognitive deficits. Approximately 10–15% of individuals with MCI progress to AD annually^2^, with a preclinical phase lasting around a decade, followed by an MCI phase of approximately four years before conversion to AD^3^.

Despite extensive efforts to identify interventions that slow or prevent AD progression, treatment options remain limited. Pharmacological approaches, including cholinesterase inhibitors, memantine, and vitamin E, have not demonstrated definitive efficacy in preventing cognitive decline^2^. Non-pharmacological strategies, such as cognitive training^4^, physical exercise^5^, and dietary modifications^6^, have shown some promise but face challenges related to adherence and scalability. These limitations underscore the need for alternative interventions that are both effective and sustainable^7^.

Transcranial direct current stimulation (tDCS) has emerged as a potential non-invasive therapy for MCI due to its accessibility, cost-effectiveness, and favorable safety profile^8^. By delivering low-intensity electrical currents through scalp electrodes, tDCS modulates cortical excitability^9^ and enhances synaptic plasticity^10^, mechanisms that are critical for cognitive function. Evidence suggests that repeated tDCS sessions may improve cognition in AD by promoting Aβ clearance, modulating blood–brain barrier integrity^11^, and increasing brain-derived neurotrophic factor (BDNF) levels^12^. Recent systematic reviews and meta-analyses indicate that tDCS is an effective tool for improving memory function in MCI^13^.

Importantly, the effectiveness of tDCS is not solely dependent on the stimulation site or duration, but also on the electrical field (EF) strength generated within the brain^14^. Higher EF strength has been linked to greater cognitive improvements in cognitively impaired individuals^15,16^. However, variability in outcomes under identical stimulation protocols suggests that individual anatomical differences influence treatment effects. Recent advances have enabled optimized, personalized tDCS, designed to enhance EF strength by incorporating individual brain structural variability^17^. A prior study found that optimizing electrode placement with simulation software increased the EF strength over the left dorsolateral prefrontal cortex (DLPFC) by 55.28% compared to traditional 10–20 EEG system placements^18^. These developments highlight EF strength as a key mediator of the clinical efficacy of tDCS, making it a critical variable for investigation in neurodegenerative conditions such as MCI.

A key biomarker for evaluating treatment efficacy in MCI and AD is white matter (WM) microstructural integrity. Diffusion tensor imaging (DTI) allows for quantitative assessment of WM integrity through metrics such as fractional anisotropy (FA), mean diffusivity (MD), and radial diffusivity (RD), which serve as early diagnostic markers and indicators of therapeutic response^19,20^. Prior studies have shown that AD patients exhibit lower FA and higher MD in key WM regions, including the splenium, fornix, and parahippocampal cingulum^21^. Aβ burden has also been linked to WM microstructural deterioration^22^, which in turn predicts the speed of conversion from normal aging to MCI^23^.

Individual AD-related factors, such as Aβ deposition, *APOE* ε4 allele status, and the *BDNF* Val66Met polymorphism, may influence the effects of tDCS on WM integrity. Aβ accumulation disrupts neuronal connectivity and synaptic function, potentially modulating neuroplastic responses to tDCS^24,25^. The *APOE* ε4 allele exacerbates Aβ-related damage, impairs synaptic plasticity, and compromises blood–brain barrier function, which may alter the neurophysiological effects of tDCS^26^. Additionally, the *BDNF* Val66Met polymorphism affects neuroplasticity, with the Met allele associated with reduced BDNF secretion, potentially diminishing tDCS-induced synaptic modulation^12,27^. Despite their significance, these individual factors remain underexplored in the context of tDCS treatment for MCI. Recent findings suggest that tDCS may influence WM integrity. Specifically, our prior research demonstrated that *APOE* ε4 carriers exhibited greater FA increases in the right uncinate fasciculus after tDCS. Additionally, Val66 homozygotes showed increased MD in the right uncinate fasciculus and decreased MD and RD in the left cingulum^28^. These results highlight the necessity of considering individual factors related to AD when evaluating tDCS efficacy.

Building upon this foundation, the present study focuses on the role of EF strength as the main predictor of tDCS-induced changes in WM microstructure. The primary aim of this study is to investigate the effects of optimized EF strength on WM microstructural integrity in MCI patients and to examine how these effects vary according to individual AD-related factors. To achieve this, we applied a two-week protocol consisting of ten consecutive tDCS sessions and assessed WM integrity changes using DTI metrics. We hypothesize that stronger EF strength will be associated with enhanced WM integrity and that this association will be modulated by AD-related individual characteristics.

## Materials and methods

### Participants

Participants were recruited from the Brain Health Center at Yeoui-do St. Mary’s Hospital, affiliated with the College of Medicine, the Catholic University of Korea. The inclusion criteria were as follows: (1) Diagnosis in accordance with Petersen’s criteria for MCI^29^, with scores below 8 on the Seoul-Instrumental Activities of Daily Living (S-IADL) scale to confirm independent functioning in daily life^30^, and (2) a Clinical Dementia Rating (CDR) score of 0.5. The exclusion criteria were: (1) A history of alcohol or drug abuse, head trauma, or psychiatric disorders; (2) use of psychotropic medications, including cholinesterase inhibitors, N-Methyl-D-Aspartate receptor antagonists, antidepressants, benzodiazepines, or antipsychotics; (3) contraindications for tDCS or MRI, such as the presence of ferromagnetic or coiled metal implants; and (4) any dermatological condition affecting scalp integrity.

Additionally, only individuals with a Hamilton Depression Rating Scale (HAMD) score of 7 or lower were included to ensure depressive symptoms were within the normal range^31^. All participants were tDCS-naive, meaning they had never previously received tDCS treatment. The selection process was supervised by two geriatric psychiatry specialists. Participants consented to a medical record review, and all assessments were conducted at the Brain Health Center, Yeoui-do St. Mary’s Hospital, the Catholic University of Korea. The study was conducted in compliance with the Declaration of Helsinki and received approval from the Institutional Review Board (IRB) of the Catholic University of Korea (SC19DEST0012). Written informed consent was obtained from all participants before their enrollment.

### Study protocol

This single-arm, prospective study was conducted without a sham control condition. Participants received ten sessions of tDCS, administered in their homes at a frequency of five sessions per week over two weeks. The ten-session protocol was selected based on previous clinical research, which demonstrated its effectiveness in treating AD and MCI^32–35^, while also considering adherence feasibility in older adults.

Neuropsychological assessments and MRI scans were performed at the Brain Health Center of Yeoui-do St. Mary’s Hospital, both within two weeks prior to the first tDCS session and after the completion of the tenth session. Additionally, participants underwent [^18^F] flutemetamol (FMM) positron emission tomography-computed tomography (PET-CT) imaging and genetic testing for *APOE* and *BDNF* variants, all completed within four weeks before the initiation of tDCS. To maintain blinding, neither the participants nor the neuropsychological examiners had access to the results of the FMM-PET, *APOE* genotyping, or *BDNF* testing.

A detailed schematic outlining the experimental procedures, previously presented in an earlier study, is available for reference^28^. This research was registered with the Clinical Research Information Service of the Korea Disease Control and Prevention Agency (KCT0006020) and was conducted from May 2020 to February 2022 at the Brain Health Center. The authors declare that they have no ethical or financial conflicts of interest related to any of the equipment manufacturers used in this study.

### Transcranial direct current stimulation application

**Fig. 1:**
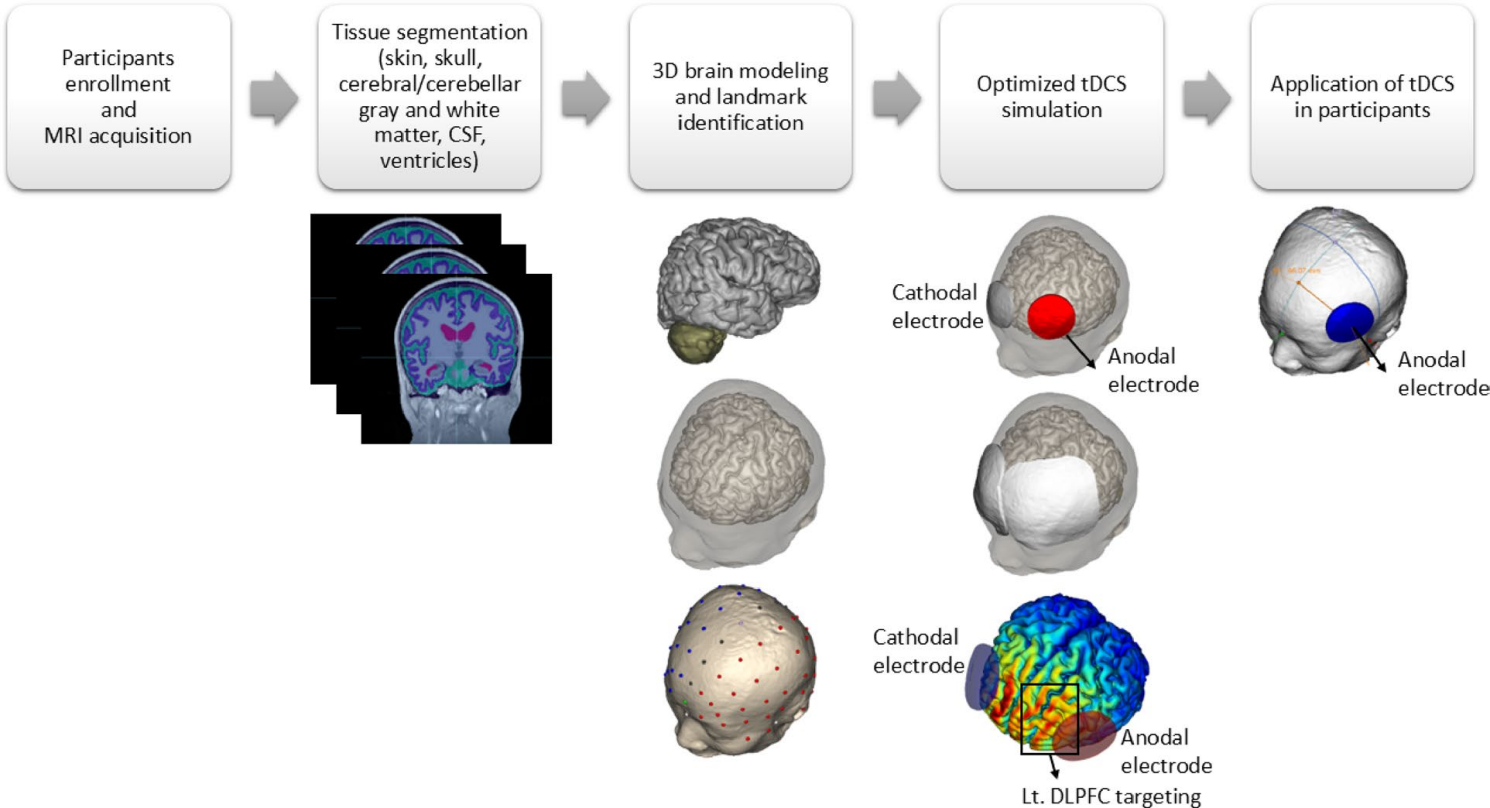
Stepwise workflow for individualized tDCS modeling and application. This figure outlines the procedure for creating and applying individualized tDCS. ① MRI acquisition from participants. ② Tissue segmentation of the MRI data. ③ 3D brain modeling from the segmented tissues. ④ Optimized tDCS simulation showing electrode placement and current flow targeting the Left Dorsolateral Prefrontal Cortex (Lt. DLPFC). ⑤ tDCS application to the participant’s scalp based on the individualized model.

In this procedure, a stable direct current of 2 mA was administered for 20 min using an MRI-compatible stimulator (YDS-301N, YBrain, Seoul, Republic of Korea). The NEUROPHET tES LAB software (version 3.0; Neurophet, Seoul, Republic of Korea) was employed to construct individualized brain models and estimate tDCS-induced EF strength. This software also determined each participant’s optimal electrode positioning based on their unique brain structure, ensuring targeted stimulation. A detailed schematic of this process is shown in Fig. 1.

To generate the brain model and analyze the tDCS-induced EF, all participants underwent baseline T1-weighted MRI scans. The software segmented these images into distinct anatomical structures, including skin, skull, cerebral gray and white matter, cerebellar gray and white matter, cerebrospinal fluid (CSF), and ventricles, creating a 3D brain reconstruction for each individual. The electrical conductivity values predefined in the software were: skin (0.465 S/m), skull (0.010 S/m), cerebral and cerebellar gray matter (0.276 S/m), cerebral and cerebellar white matter (0.126 S/m), and CSF/ventricles (1.65 S/m)^36^. After the brain model was generated, an investigator assigned anatomical landmarks (nasion, inion, and preauricular points) to facilitate accurate electrode placement.

The stimulation was directed at the DLPFC, with the anode positioned over the left DLPFC and the cathode placed over the contralateral supraorbital region. This montage is a widely used configuration in prefrontal tDCS, but it’s important to note that the supraorbital site is not functionally inert, as it overlies the frontopolar cortex (FPC). Therefore, throughout the paper we interpret our findings as effects of the paired DLPFC-FPC montage rather than attributing them solely to the active electrode. Disk-shaped electrodes with a 3 cm radius were used. The tDCS intensity (2 mA) was input into the software, which then computed optimized electrode locations by simulating the EF distribution across the participant’s brain. The software systematically adjusted electrode positions around the target region to identify the placement that maximized the EF strength induced by tDCS. The software then generated precise placement guides, allowing trained personnel to correctly position the electrodes.

The stimulation was administered by trained staff, who conducted home visits for each session. The electrode positions were measured using distances from preauricular points to the electrode center, aligning with a reference line extending from the vertex to the nasion. Before each session, the electrode positioning was carefully verified using these anatomical landmarks. Additionally, 15 min into the session, staff reassessed electrode positioning to ensure accuracy. Each participant was consistently monitored by the same staff member across all ten sessions.

For each participant, the individualized EF distribution was quantitatively assessed using the peak EF strength (V/m) within the left DLPFC target region. This optimized EF strength value was extracted and used as a continuous variable for subsequent statistical analysis to examine its relationship with changes in WM microstructural integrity.

### Neuropsychological assessment

All participants underwent cognitive assessments utilizing the Korean adaptation of the Consortium to Establish a Registry for Alzheimer’s Disease (CERAD-K)^37^. This battery included the Korean versions of several tests: Verbal Fluency (VF), the 15-item Boston Naming Test, and the Korean version of the Mini-Mental State Examination (MMSE-K)^38^, along with assessments for word list memory (WLM), recall, recognition, constructional praxis, and constructional recall (CR). The comprehensive CERAD-K score was calculated as the sum of all test scores, excluding the MMSE-K and CR.

To assess executive function, we employed the Korean Stroop Word-Color Test (K-SWCT), which measures response inhibition in both letter and color reading tasks^39^, and the Trail Making Test B (TMT-B), which evaluates processing speed and cognitive flexibility by measuring the time required to connect numbers and letters in sequential order. A detailed description of these assessments is available in the Supplementary Material.

### MRI acquisition and processing

Detailed information regarding MRI acquisition and processing is available in the Supplementary Material.

### Processing procedures of the DTI images

Details of the MRI acquisition procedures are provided in the Supplementary Material. The imaging data were preprocessed using Statistical Parametric Mapping 12 (SPM12) (https://www.fil.ion.ucl.ac.uk/spm/software/spm12), running on MATLAB version 2018b, along with the PANDA toolbox (https://www.nitrc.org/projects/panda/) and the FMRIB Software Library (FSL) version 6.0 (https://fsl.fmrib.ox.ac.uk/fsl/fslwiki/FSL).

The PANDA processing pipeline consisted of two primary stages: (1) preprocessing and (2) generating diffusion metrics. The preprocessing workflow included the following five steps: (1) estimating the brain mask, (2) cropping raw images, (3) correcting for eddy-current distortions, (4) averaging multiple acquisitions, and (5) computing diffusion tensor (DT) metrics. The DT metrics analyzed in this study included FA, MD, and RD. The resulting diffusion metric images were then normalized to the Montreal Neurological Institute (MNI) standard space for further analysis.

To facilitate regional analysis, diffusion metric images with a voxel size of 1.0 × 1.0 × 1.0 mm^3^ in standard space were processed using a WM probabilistic tract atlas. This atlas consists of 20 WM tracts, which were identified probabilistically through deterministic tractography performed on a cohort of 28 healthy individuals^40^. The WM probabilistic tract atlas includes the following WM tracts: (1) anterior thalamic radiation (ATR), (2) cingulum in the cingulate cortex, (3) cingulum in the hippocampal area, (4) corticospinal tract (CST), (5) forceps major, (6) forceps minor, (7) inferior fronto-occipital fasciculus (IFOF), (8) superior longitudinal fasciculus (SLF), (9) temporal projection of the SLF, (10) inferior longitudinal fasciculus (ILF), and (11) uncinate fasciculus (UF).

For each WM tract, separate statistical results were obtained for the left and right hemispheres, except for the forceps major and minor, which were analyzed as a unified region. This procedure yielded 20 tracts in total. These statistical files contained values for FA, MD, and RD, each of which provides distinct insights into WM integrity^41^:

1. FA (Fractional Anisotropy): Represents the degree of directional water diffusion within a voxel. Higher FA values suggest greater microstructural integrity of WM.
2. MD (Mean Diffusivity): Reflects the overall magnitude of water diffusion, irrespective of direction. Higher MD values indicate increased water mobility, which may suggest lower WM density, structural degradation, or potential injury.
3. RD (Radial Diffusivity): Measures water diffusion perpendicular to the principal direction. Increased RD values are often interpreted as markers of demyelination, providing insight into myelin integrity.

Additional details on the PANDA processing pipeline can be found in a previous study^42^.

### Aβ deposition

The methodology for acquiring and processing [^18^F]-FMM PET images and calculating standardized uptake value ratios (SUVRs) to quantify Aβ deposition are detailed in the Supplementary Material. A threshold of 0.62 was employed to differentiate between Aβ positive (Aβ +) and Aβ negative (Aβ -) accumulations, aligning with previous FMM-PET studies^43^. It is crucial to note that ‘negative accumulation’ denotes subthreshold deposition rather than a complete absence of amyloid deposition.

### *APOE* genotyping

The approach for *APOE* genotyping is detailed in the Supplementary Material. According to our protocol, we would exclude subjects who possessed the *APOE* ε2 allele due to its observed protective role^44^. Participants were classified based on the presence of the *APOE* ε4 allele; those with at least one ε4 allele were grouped as *APOE* ε4 carriers, whereas those without any ε4 alleles were designated as non-carriers.

### *BDNF* genotyping

The methodology for *BDNF* genotyping is described in the Supplementary Material. In the context of the *BDNF* Val66Met polymorphism (rs6265), we categorized participants based on existing genetic research^45,46^ into groups: those carrying at least one Met66 allele were labeled as Met carriers, whereas those without any Met66 alleles were considered Met non-carriers.

### Statistical analysis

Statistical analyses were performed using R software (version 4.3.0), jamovi (version 2.6.19), and SPM 12 (https://www.fil.ion.ucl.ac.uk/spm/). The normality of continuous variables was assessed using the Kolmogorov–Smirnov test, and data were standardized using z-score transformation prior to analysis.

Partial correlation analyses were conducted to investigate associations between optimized EF strength and changes in WM integrity metrics, including FA, MD, and RD. These analyses adjusted for covariates such as age, sex, years of education, Aβ deposition, *APOE* ε4 carrier status, and *BDNF* Val66Met polymorphism. To address multiple comparisons across DTI metrics and tracts, false discovery rate (FDR) correction was applied using the Benjamini–Hochberg procedure, with a significance threshold of q < 0.05. Both unadjusted and FDR-adjusted *P*-values are reported to ensure comparability with prior literature and to account for potential type I error. Additionally, correlation analyses using Pearson’s product-moment correlation were performed to examine relationships between changes in FA across tracts of interest identified as relevant to optimized EF strength.

Based on the findings from these partial correlation analyses, correlation analyses using Pearson’s product-moment correlation were performed to evaluate the relationships between changes in DTI parameters within the identified tracts of interest and changes in total and domain scores of the CERAD-K. In addition, exploratory paired-sample t-tests were performed to compare pre- and post-intervention scores for cognitive assessments and DTI metrics (FA, MD, and RD).

Multiple regression analyses were applied to examine the interaction effects of optimized EF strength and individual modifiers (e.g., Aβ deposition, *APOE* ε4 carrier status, and *BDNF* polymorphism) on WM integrity metrics. Additional models were conducted to investigate whether these interaction effects extended to cognitive outcomes, testing the interaction between optimized EF strength and each modifier for changes in CERAD-K total and domain scores. Covariates included age, sex, years of education, and the effect modifiers not included in the specific interaction model under evaluation.

For interaction analyses, unadjusted *P*-values were considered the primary results because the models were hypothesis-driven and limited to a small number of predefined effect modifiers, thereby reducing the risk of inflated type I error. Nonetheless, FDR correction (Benjamini–Hochberg) was additionally applied to interaction terms with nominal significance (*P* < 0.05) within each metric category (FA, MD, RD) and for cognitive outcomes, with q < 0.05 indicating statistical significance. All statistical analyses were two-tailed, and significance thresholds were set as described above.

## Results

### Baseline demographic and clinical data

**Fig. 2:**
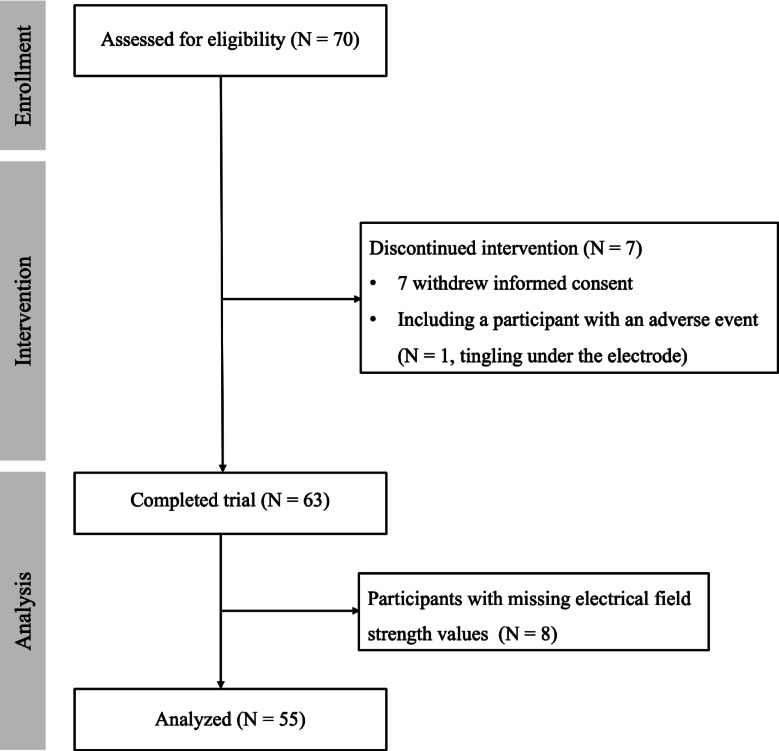
The flowchart of the study.

A total of 70 participants were assessed for eligibility. Of these, 63 completed the intervention and post-tDCS assessments. Seven participants discontinued the intervention: six withdrew informed consent, and one experienced a mild adverse event (tingling under the electrode). Among the 63 participants who completed the intervention, eight were excluded from the analysis due to missing EF strength values. Therefore, the final analyses were conducted on 55 participants. Figure 2 provides a detailed flowchart of participant recruitment, retention, and inclusion. Table 1 shows the baseline demographic data for the participants who completed the study. Post-intervention, significant improvements were observed in several CERAD-K domains (e.g., BNT, WLM, WLRc, Stroop word–color) and in the total CERAD-K score, while other domains showed no significant changes. The number and distribution of participants based on individual factors are described in the Supplementary Material.

**Table 1:** Baseline demographic and clinical characteristics of the study participants.

Demographic and clinical characteristics (N = 55)				
Age (years)	72.7 ± 8.2			
Sex				
-Male	17 (30.9%)			
-Female	38 (69.1%)			
Years of education	12.2 ± 4.9			
[^18^F] Flutemetamol deposition (positivity, %)	20 (36.4%)			
Global [^18^F] Flutemetamol SUVR_PONS_	0.61 ± 0.15			
*APOE* ε4 carrier status (carrier. %)	26 (47.3%)			
*BDNF* polymorphism (Val/Met or Met/Met, %)	45 (81.8%)			
CERAD-K	Pre-intervention	Post-intervention	t	p
VF	11.9 ± 5.2	12.1 ± 5.4	0.50	0.619
BNT	10.7 ± 3.2	11.3 ± 3.2	2.88	0.006
MMSE	23.1 ± 5.2	23.1 ± 5.6	-0.13	0.900
WLM	14.4 ± 4.8	16.4 ± 5.7	4.80	0.000
CP	10.1 ± 1.6	10.1 ± 1.6	0.14	0.890
WLR	3.8 ± 2.6	4.0 ± 3.1	0.70	0.486
WLRc	6.8 ± 2.9	7.6 ± 2.4	2.41	0.019
CR	4.6 ± 3.5	4.8 ± 3.9	0.68	0.498
TMT B	223.1 ± 79.1	222.4 ± 95.6	-0.07	0.942
Stroop word-color	25.9 ± 14.0	28.2 ± 13.9	2.31	0.025
Total CERAD-K	57.7 ± 16.1	61.4 ± 17.3	4.70	0.000
Pre-optimization EF strength (V/m)	0.19 ± 0.07			
Optimized EF strength (V/m)	0.24 ± 0.07			

Data are presented as the mean ± SD unless indicated otherwise. BNT, Boston Naming Test; CERAD-K, Korean version of Consortium to Establish a Registry for Alzheimer’s Disease; CP, Constructional Praxis; CR, constructional recall; EF, electrical field; MMSE, the Korean version of the Mini-Mental Status Examination; SUVR_PONS_, standardized uptake value ratio of [^18^F] Flutemetamol, using the pons as a reference region; TMT B, Trail Making Test B; Total CERAD-K, composite score summing scores of the CERAD-K VF, BNT, WLM, CP, WLR, and WLRc domains; VF, verbal fluency; WLM, Word List Memory; WLR, Word List Recall; WLRc, Word List Recognition.

### Changes in WM microstructural integrity and optimized EF strength

Simulation-based optimization of electrode positioning yielded individualized EF distributions over the left DLPFC. On average, optimized placement increased EF strength from 0.187 ± 0.065 V/m to 0.244 ± 0.072 V/m (Δ = 0.057 V/m, 95% CI 0.047–0.067), corresponding to a 30.4% improvement relative to conventional 10–20 EEG-based placement.

**Fig. 3:**
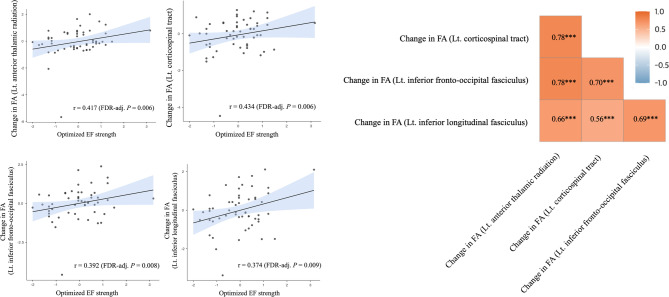

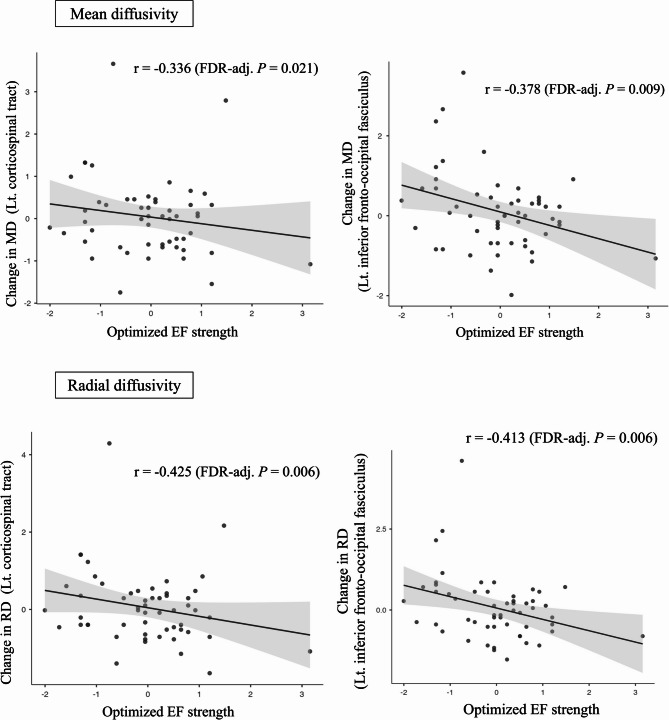
Associations between optimized electrical field strength and changes in white matter microstructural integrity after 10 sessions of anodal tDCS. (**A**) Fractional anisotropy. (**B**) Mean diffusivity; Radial diffusivity. Partial correlation analysis adjusting for age, sex, education years, Aβ deposition, *APOE* ε4 carrier status, and BDNF polymorphism. (**A**) Right panel: A partial correlation analysis examining the pairwise associations among changes in fractional anisotropy of the white matter tracts of interest. The r-values are from Pearson’s product-moment correlation, and *p*-values were adjusted for multiple comparisons using the Benjamini–Hochberg False Discovery Rate method; ***, *P* < 0.001. FA, fractional anisotropy; MD, mean diffusivity; RD, radial diffusivity.

After 10 sessions of anodal tDCS, significant associations were observed between optimized EF strength and changes in WM integrity metrics, including FA, MD, and RD. Partial correlation analyses, adjusted for age, sex, education years, Aβ deposition, *APOE* ε4 carrier status, and *BDNF* Val66Met polymorphism, demonstrated significant correlations between optimized EF strength and changes in these metrics across tracts of interest (Fig. 3).

For FA, significant positive correlations were identified in the following tracts: left ATR (r = 0.417, unadjusted *P* = 0.003, FDR-adjusted *P* = 0.006), left CST (r = 0.434, unadjusted *P* = 0.002, FDR-adjusted *P* = 0.006), left IFOF (r = 0.392, unadjusted *P* = 0.005, FDR-adjusted *P* = 0.008), and left ILF (r = 0.374, unadjusted *P* = 0.008, FDR-adjusted *P* = 0.009). Significant correlations were identified between changes in FA across the WM tracts of interest (Fig. 3A). As shown in the correlation matrix (Fig. 3, right panel), all pairwise correlations among changes in the left CST, left IFOF, and left ILF were statistically significant (all *P* < 0.001), indicating consistent positive associations across these tracts.

For MD and RD, significant negative correlations were observed in the same tracts of interest. Specifically, the correlations for MD and RD were as follows: left CST (r = -0.336 and − 0.425, respectively; unadjusted *P* = 0.018 and 0.002, FDR-adjusted *P* = 0.021 and 0.006), and left IFOF (r = − 0.378 and − 0.413, respectively; *P* = 0.007 and 0.003, FDR-adjusted *P* = 0.009 and 0.006) (Fig. 3B). However, no significant associations were observed between optimized EF strength and changes in WM integrity metrics in other WM tracts included in the probtract atlas.

Additionally, correlation analyses using Pearson’s product-moment correlation did not reveal any significant associations between changes in DTI parameters within the identified tracts of interest and changes in total and domain scores of the CERAD-K.

### Interactions between optimized EF strength and individual factors associated with AD

**Fig. 4:**
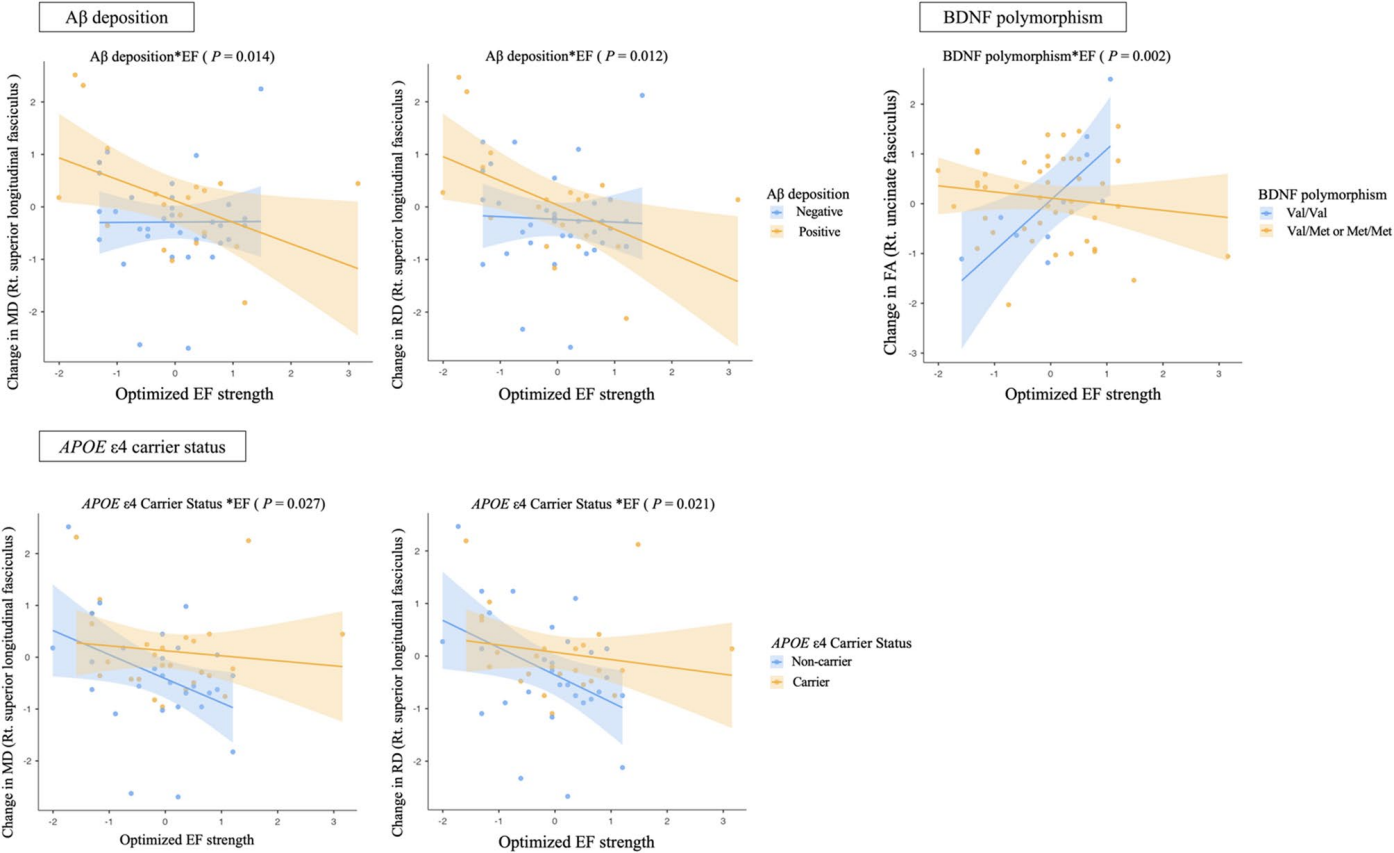
Associations between optimized electrical field strength and changes in white matter microstructural integrity according to Aβ deposition, *APOE* ε4 carrier status, and BDNF polymorphism. Multiple regression analysis was used to predict the impact of effect modifier-by-optimized electrical field strength (effect modifier*EF) for white matter microstructural integrity (effect modifiers: Aβ deposition, *APOE* ε4 carrier status, and BDNF polymorphism), adjusting for age, sex, education years, and effect modifier not included in each interaction evaluation. FA, fractional anisotropy; MD, mean diffusivity; RD, radial diffusivity.

Significant associations were identified between optimized EF strength and changes in WM microstructural integrity, with these effects varying based on individual effect modifiers (Fig. 4). Notably, Aβ deposition moderated the relationship between EF strength and microstructural changes in the right SLF (MD: β =  − 0.705, 95% confidence interval [CI] − 1.262 to − 0.148, *P* = 0.014; RD: β =  − 0.731, 95% CI − 1.289 to − 0.172, *P* = 0.012). Specifically, increased EF strength was associated with reductions in MD and RD in Aβ-positive patients. For MD (values are presented as × 10⁻3), the Aβ-negative group showed a decrease from 0.841 to 0.838 [t(34) =  − 1.261, *P* = 0.216], whereas the Aβ-positive group showed an increase from 0.844 to 0.850 [t(19) = 2.088, *P* = 0.050]. For RD (values are presented as × 10⁻3), the Aβ-negative group decreased from 0.696 to 0.695 [t(34) =  − 0.622, *P* = 0.538], whereas the Aβ-positive group increased from 0.698 to 0.703 [t(19) = 2.054, *P* = 0.054].

In addition, a significant interaction was observed between EF strength and *APOE* ε4 carrier status in predicting WM microstructural changes in the right SLF (MD: β = 0.641, 95% CI 0.077 to 1.206, *P* = 0.027; RD: β = 0.673, 95% CI 0.108 to 1.239, *P* = 0.021). Specifically, EF strength was associated with greater reductions in MD and RD among non-carriers compared to carriers. For MD (values are presented as × 10⁻3), the non-carrier group showed a decrease from 0.851 to 0.849 [t(28) =  − 0.670, *P* = 0.508], while the carrier group showed an increase from 0.832 to 0.835 [t(25) = 0.950, *P* = 0.351]. For RD (values are presented as × 10⁻3), the non-carrier group decreased from 0.708 to 0.707 [t(28) =  − 0.212, *P* = 0.834], whereas the carrier group increased from 0.685 to 0.688 [t(25) = 1.196, *P* = 0.243].

Additionally, BDNF polymorphism influenced the relationship between EF strength and WM integrity (β =  − 1.178, 95% CI − 1.912 to − 0.444, *P* = 0.002). Higher EF strength was associated with increased FA in the right UF among Met allele non-carriers. In the Met non-carrier group, FA in the right UF increased from 0.322 to 0.327 [t(9) = 1.158, *P* = 0.277]. In the Met carrier group, FA in the right UF decreased from 0.323 to 0.322 [t(44) =  − 0.619, *P* = 0.539].

**Fig. 5:**
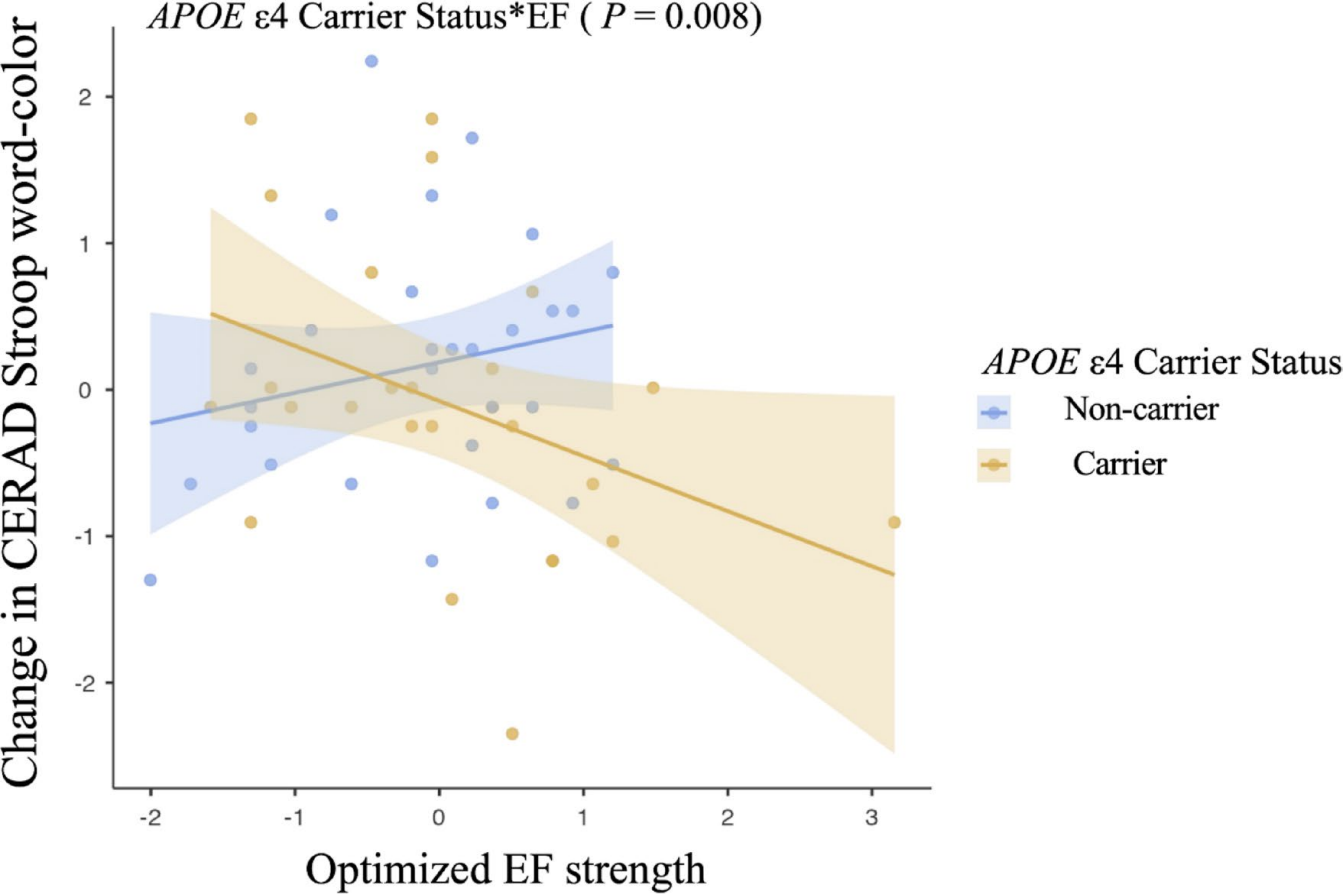
Associations between optimized electrical field strength and changes in cognitive function according to *APOE* ε4 carrier status. Multiple regression analysis was used to predict the impact of effect modifier-by-optimized electrical field strength (effect modifier*EF) for cognitive function (effect modifiers: Aβ deposition, *APOE* ε4 carrier status, and BDNF polymorphism), adjusting for age, sex, education years, and effect modifier not included in each interaction evaluation.

Regarding cognitive outcomes, interaction analyses revealed that APOE ε4 status moderated the relationship between EF strength and cognitive change (Fig. 5). Specifically, a significant interaction was found for the CERAD SWCT (β =  − 0.708, 95% CI − 1.128 to − 0.198, *P* = 0.008), indicating that higher EF strength was associated with improved Stroop performance in non-carriers, whereas carriers showed no improvement or a decline. In the paired comparisons, non-carriers demonstrated a significant increase in Stroop Color-Word scores from 25.4 to 28.5 [t(28) = 2.647, *P* = 0.013], whereas carriers showed a smaller, non-significant increase from 26.6 to 27.8 [t(25) = 0.762, *P* = 0.453]. No significant interactions were observed between EF strength and *APOE* ε4 status for the CERAD-K total score or other domain scores.

For interaction terms that reached nominal significance in unadjusted analyses, the FDR-adjusted *P*-values were as follows: Aβ × EF in the right SLF (MD, q = 0.986; RD, q = 0.961), *APOE* ε4 × EF in the right SLF (RD, q = 0.961; MD, q = 0.986), BDNF × EF in the right UF (FA, q = 0.180), and *APOE* ε4 × EF in the SWCT (q = 0.281).

## Discussion

This study investigated the effects of two weeks of consecutive tDCS on WM microstructural integrity in individuals with MCI and examined how these effects varied according to individual factors related to AD, including Aβ deposition, *APOE* ε4 carrier status, and *BDNF* Val66Met polymorphism.

We observed significant associations between optimized EF strength and WM integrity changes. Higher EF strength was linked to greater FA increases and reduced MD and RD in left-lateralized tracts, including the ATR, CST, IFOF, and ILF. These findings suggest that a greater EF strength may facilitate more robust WM plasticity, potentially by increasing axonal coherence and myelination^47^. Previous studies have reported tract-specific FA increases following tDCS, such as in the CST of stroke patients and in the UF of individuals with MCI^28,48^. In addition, tDCS combined with cognitive training has been shown to induce microstructural plasticity in older adults, where greater behavioral improvements were associated with a significant decrease in gray matter MD in the stimulated temporooccipital region^49^. Extending these findings, our results demonstrate that individually optimized EF strength is a key determinant of such WM plasticity, linking FA increases and MD/RD reductions to the spatial distribution of stimulation.

Each affected tract plays a distinct role in cognitive and neural function. The ATR is critical for executive function and working memory, and its integrity has been associated with cognitive recovery in individuals with MCI^50,51^. The left CST, primarily responsible for motor function^52^, showed the strongest correlation with EF strength, suggesting that optimized tDCS may exert effects on motor-related pathways. The left IFOF and ILF, involved in visuospatial processing^53^ and memory function^54^, respectively, also exhibited EF strength-dependent changes. Furthermore, the current study identified significant correlations among FA changes across these tracts. This suggests that tDCS may have induced synchronized structural changes across these pathways. It is plausible that as EF strength increased, axonal coherence and myelination improved concurrently across these tracts. Previous studies have reported synchronized WM changes following tDCS, particularly in pathways such as the CST and IFOF, which may contribute to network-level neuroplasticity and functional integration^55,56^. These findings highlight the potential for tDCS to influence broader cognitive networks rather than exerting purely localized cortical effects, emphasizing the importance of optimizing EF strength to enhance network-level plasticity while avoiding potential overstimulation effects.

Beyond FA changes, significant reductions in MD and RD were found in the left CST and left IFOF. Lower MD and RD values indicate reduced extracellular diffusion, typically associated with improved axonal integrity and myelination^57,58^. Given that MD and RD are sensitive markers of demyelination and overall tissue integrity, these findings suggest that tDCS may contribute to structural stabilization or repair. The specificity of these changes in certain tracts may be attributed to their proximity to the stimulation site or differential sensitivity to tDCS-induced plasticity. The left IFOF and CST, which are closer to the DLPFC stimulation site, exhibited more pronounced effects, whereas deeper tracts such as the ATR and ILF showed less change. As EF strength is strongest at the cortical surface and diminishes with depth^59^, these variations may reflect structural and functional differences in tract responsiveness to tDCS. Future studies should explore how variations in EF strength and electrode configuration influence WM integrity across different pathways.

Furthermore, it is imperative to consider the specific electrode montage when interpreting the findings. The contralateral supraorbital return corresponds to the FPC, which is not functionally inert. Prior studies have shown that tDCS over the FPC can induce widespread, frequency-dependent network changes and alter behavior^60–62^. Consistent with this, a recent clinical trial for multiple sclerosis explicitly employed a DLPFC-FPC montage to engage cognitive-affective circuitry, highlighting that the return site actively shapes the induced effects and should not be treated as a neutral reference^63^. Modeling work further supports this view, showing that the return electrode influences EF strength and distribution. Thus, tDCS effects should be understood as arising from the paired DLPFC–FPC configuration rather than the DLPFC alone^64,65^, underscoring the need for careful interpretation of montage-dependent effects in future studies.

The interpretation of EF strength in a clinical context warrants further investigation. While greater EF strength was associated with more robust WM integrity in this study, we did not identify a minimal effective threshold or evidence of a saturation effect within the observed intensity range^66^. As the EF values were optimized individually and remained within a relatively narrow distribution, future research using a broader stimulation intensity spectrum will be necessary to determine whether nonlinear effects or physiological thresholds exist. Additionally, considerable interindividual variability in EF distribution is likely influenced by anatomical factors such as skull thickness, scalp-to-cortex distance, and the degree of brain atrophy^67,68^. These structural differences can alter both the focality and magnitude of current delivery, particularly in older adults or individuals with neurodegeneration, and should be considered in the development of personalized stimulation protocols.

The influence of individual AD-related factors was evident in this study. In Aβ-positive individuals, higher EF strength correlated with greater MD and RD reductions in the right SLF, suggesting that tDCS may promote compensatory plasticity in regions with Aβ pathology. The SLF is a key pathway linking the frontal and parietal lobes and plays a critical role in working memory and executive function^69^. Previous studies have reported FA reductions in the SLF in AD patients, linking such changes to cognitive decline^70^. Our findings suggest that tDCS may enhance WM integrity in Aβ-positive individuals, potentially mitigating structural deterioration associated with AD pathology.

Similarly, *APOE* ε4 carrier status significantly modulated the effects of tDCS. Non-carriers exhibited greater MD and RD reductions in the right SLF compared to carriers, indicating that *APOE* ε4 allele may attenuate the neuroplastic effects of tDCS. *APOE* ε4 allele is a well-established genetic risk factor for AD, and prior studies have shown that carriers experience more severe WM damage and reduced neuroplastic potential^71,72^. In addition, a significant interaction between optimized EF strength and *APOE* ε4 status was observed in relation to executive function, as measured by the CERAD-K SWCT. Specifically, higher EF strength was associated with greater improvement in Stroop performance among non-carriers compared with carriers. Given that the SLF is involved in executive function via frontoparietal connectivity, EF strength-related improvements in WM integrity within the right SLF likely contributed to the cognitive gains observed in non-carriers. These findings indicate a potential structure–function relationship whereby optimized EF strength may facilitate both WM plasticity and functional gains in individuals without the *APOE* ε4 allele. Therefore, *APOE* ε4 carriers may require adjusted stimulation protocols to achieve comparable neuroplastic and cognitive benefits.

*BDNF* Val66Met polymorphism also influenced FA changes, with non-carriers of the Met allele exhibiting greater FA increases in the right UF. The UF is a major tract connecting the prefrontal cortex and temporal lobe and is essential for memory and emotional regulation^73^. Reduced FA in the UF has been reported in AD and is associated with disease progression^74^. Given that BDNF plays a central role in neuroplasticity^75^ and that the Met allele is associated with reduced BDNF secretion^12,76^, our findings suggest that BDNF-mediated mechanisms influence the neurophysiological response to tDCS. These results underscore the relevance of genetic factors in designing personalized neuromodulation approaches.

However, when considering the robustness of these moderator effects, several caveats should be noted. In interpreting the moderator analyses, it should be noted that none of the interaction terms remained significant after FDR correction. Although these results were based on a hypothesis-driven approach with predefined modifiers, the modest sample size and inherent variability within the MCI cohort likely reduced statistical power. Therefore, the observed nominal interactions should be regarded as exploratory and interpreted with caution, representing biologically plausible yet preliminary trends rather than definitive evidence. While these findings provide initial support for the hypothesis that individual AD-related factors influence EF-related WM plasticity and cognitive outcomes, confirmation in larger, sham-controlled studies is essential to clarify whether these moderator effects reflect true structure–function relationships and to establish the clinical utility of tailoring tDCS protocols to individual genetic and pathological profiles.

Against this background, it is also noteworthy that the EF strength–related WM changes identified here were not directly accompanied by corresponding improvements in cognitive performance when analyzed within those EF-sensitive tracts within the two-week protocol. These findings suggest that, although optimized EF strength may induce physiologically meaningful WM plasticity, such structural modifications may not immediately translate into short-term cognitive benefits in a tract-specific manner.

In contrast, previous findings from the same cohort reported tDCS-induced cognitive improvements in specific subgroups, including Aβ-negative individuals and those without the *BDNF* Met allele^28,77^. Additionally, prior analyses showed that baseline Aβ burden was significantly associated with FA in the left hippocampal cingulum, which in turn predicted delayed memory improvement^28^. This highlights a critical distinction: while certain WM tracts, including the left hippocampal cingulum, are linked to both AD pathology and cognitive outcomes, the EF-sensitive tracts identified in the present study did not show such associations. This dissociation suggests that EF strength-related WM plasticity may influence cognitive outcomes through longer-term or more distributed network-level mechanisms, which may not be fully captured within the short duration of the current study. In line with this interpretation, a plausible mechanistic account can be proposed to explain how such EF-related WM changes might eventually translate into cognitive benefits. A mechanistic interpretation consistent with our findings is that EF strength fosters network-level compensation within frontoparietal systems rather than producing immediate tract-specific behavioral gains. First, coordinated FA increases and concomitant reductions in MD and RD suggest microstructural processes compatible with axonal coherence and myelin-related stabilization, which can improve conduction timing and synchrony but may require extended intervals or task engagement to yield measurable performance changes^57,58^. Second, tDCS can modify large-scale functional connectivity and promote synchronized pathway remodeling, indicating distributed rather than purely focal effects that may manifest as delayed or context-dependent cognitive benefits ^55,56^. Third, the SLF supports executive control within frontoparietal networks; WM alterations within this pathway have been linked to working memory and executive performance in AD and related conditions, which supports the moderator-dependent Stroop findings observed here while also explaining the absence of uniform, short-term effects across all tracts^21,23,69,70^. Taken together, these considerations provide a biologically plausible account for EF-related WM integrity that precedes or outlasts the time window for detectable cognitive change in this study.

Several limitations should be acknowledged. First, the absence of a sham or traditional tDCS control group, as well as a healthy control group, is a notable limitation. The study employed a single-arm design to prioritize statistical power in evaluating within-subject associations between optimized EF strength and white matter changes across multiple AD–related individual factors. Including additional control arms would have required a substantially larger sample size, which was not feasible within the logistical scope of this study and would have compromised statistical power. Furthermore, inclusion of a sham group would have required delayed provision of active stimulation for ethical reasons, and such a crossover protocol was not feasible within the logistical constraints of the study. While this limits causal inference regarding the efficacy of stimulation itself, the design was appropriate for the exploratory objective of identifying moderators of neuromodulatory response in a clinically defined MCI population. Second, the two-week assessment period imposes limitations on the inferences about durability and clinical translation. WM microstructural changes can precede behavioral gains; therefore, longer follow-up periods (e.g., 3–6 months) are required to determine whether the observed DTI effects persist and map onto sustained cognitive improvement. Third, alternative electrode montages were not examined (e.g., bilateral DLPFC or extracephalic return). The supraorbital/frontopolar site is functionally active, and return-electrode placement is critical in shaping EF strength and distribution. Future studies should directly compare DLPFC–FPC with other montages to disentangle site-specific effects. Finally, given the interindividual variability in response to stimulation, future studies should further explore optimal EF thresholds and stimulation parameters tailored to individual characteristics.

In conclusion, this study confirms that optimized EF strength is significantly associated with WM microstructural changes in individuals with MCI. It also emphasizes the importance of considering individual AD-related factors when evaluating the efficacy of tDCS. These findings support the development of personalized neuromodulation approaches and highlight the potential of precision medicine in non-invasive brain stimulation strategies for the prevention and management of AD.

## Supplementary Information

Below is the link to the electronic supplementary material.

Supplementary Material 1

## Data Availability

The datasets generated or analyzed during the current study are not publicly available due to the Patient Data Management Protocol of Yeouido Saint Mary’s Hospital but are available from the corresponding author upon reasonable request.
