# Supplementary material for: Alzheimer-related individual factors modulate effects of transcranial direct current stimulation strength on white matter integrity in mild cognitive impairment
Source: Sci Rep. 2025 Dec 17;15:43985. doi: 10.1038/s41598-025-27612-7 (PMC12711906; doi:10.1038/s41598-025-27612-7)
Supplement: Supplementary file 1 — Supplementary Material 1 [file 41598_2025_27612_MOESM1_ESM.pdf]

Alzheimer-Related Individual Factors Modulate Effects of Transcranial Direct Current Stimulation Strength on White Matter Integrity in Mild Cognitive Impairment

Jung-Won Lee[a], Sunghwan Kim[b], Suhyung Kim[c], Yoo Hyun Um[c], Sheng-Min Wang[b], TaeYeong Kim[d], Donghyeon Kim[d], Hyun Kook Lim[b,d], Chang Uk Lee[a], Dong Woo Kang[a]*

[a]Department of Psychiatry, Seoul St. Mary's Hospital, College of Medicine, The Catholic University of Korea, Seoul, Republic of Korea

[b]Department of Psychiatry, Yeouido St. Mary's Hospital, College of Medicine, The Catholic University of Korea, Seoul, Republic of Korea

[c]Department of Psychiatry, St. Vincent's Hospital, College of Medicine, The Catholic University of Korea, Seoul, Republic of Korea

[d]Research Institute, NEUROPHET Inc.; Seoul 06247, Republic of Korea

*Address correspondence to Dong Woo Kang, MD, PhD

Department of Psychiatry, Seoul St. Mary's Hospital, College of Medicine, The Catholic University of Korea, 222, Banpo-daero, Seocho-gu, Seoul, 06591, Republic of Korea

Tel: +82-2-2258-6084, Fax: +82-2-594-3870, E-mail: kato7@hanmail.net

1 Supplementary Methods

1.1 Neuropsychological evaluation

Cognitive status was assessed using neuropsychological testing at Yeouido St. Mary's Hospital, The Catholic University of Korea. Cognitive functions in all the subjects were assessed using the Korean version of the Consortium to Establish a Registry for Alzheimer's Disease (CERAD-K), which included verbal fluency (VF), the 15-item Boston Naming Test (BNT), MMSE-K, Word List Memory (WLM), Word List Recall (WLR), Word List Recognition (WLRc), Constructional Praxis (CP), and Constructional Recall (CR). The CERAD is a standardized clinical and neuropsychological assessment battery for the evaluation of patients with AD. The results were reviewed by a neuropsychologist to determine whether there was evidence of cognitive impairment.

The VF score is the number of animal names that the subject could name in one minute. The BNT scores ranged from 0 to 15 points. The MMSE-K score ranged from 0 to 30 points. The WLM scores ranged from 0 to 30 points. The WLR scores ranged from 0 to 10 points. The WLR scores ranged from 0 to 10 points. The WLRc scores ranged from 0 to 10 points. The CP scores ranged from 0 to 11 points. The CR scores ranged from 0 to 11 points. The total memory domain scores ranged from 0 to 67 points. Finally, the total CERAD-K scores ranged from 0 to 100 points.

To evaluate executive function, we performed the Stroop task, which requires pre-directed reactions while suppressing the dominant response, such as letter reading and color reading conditions. In the current study, the Korean Stroop Word-Color Test (K-WCST) was used. The subjects were requested to read the color of letters when letters were written in red, blue, yellow, and black colors within a limited time [1]. Additionally, participants carried out trail-making test B, which alternatively

measures the time required to connect letters and numbers in the sequence. Finally, the VF test, the subdomain of the CERAD-K battery, counts the number of animals recalled within a minute.

1.2 Magnetic Resonance Imaging Acquisition

Imaging data were collected by the Department of Radiology of Yeouido Saint Mary's Hospital at the Catholic University of Korea using a 3-T Siemens Skyra MRI machine and a 32-channel Siemens head coil (Siemens Medical Solutions, Erlangen, Germany). The scanning parameters of the T1-weighted three-dimensional magnetization-prepared rapid gradient echo sequences were as follows; echo time (TE)=2.6 ms, repetition time (TR)=1,940 ms, inversion time (TI)=979 ms, flip angle (FA)=9o, field of view=250×250 mm, matrix=256×256, and voxel size=1.0×1.0×1.0 mm^3. Fluid attenuated inversion recovery (FLAIR) MRI sequences were as follows: TE= 135 ms; TR= 9000 ms; TI= 2,200 ms; FA=90o; FOV= 220x220 mm; matrix=356x231; and voxel size=1.0x 1.0x1.0 mm^3. In addition, the scanning parameters of the DTI sequences were as follows; echo planar imaging, TR=3,100 ms, TE=86 ms, field of view=224 mm, voxel dimension=2 mm isotropic, B-value=1,000, gradients applied=64 isotropically, and distributed and acquisition time=5 min 44sec.

1.3 *APOE* genotyping

DNA was isolated from blood using the QIAmp Blood DNA Maxi Kit protocol (Qiagen, Valencia, CA). Genotypes for two APOE SNPs, rs429358 (E*4) and rs7412 (E*2) were determined using TaqMan SNP genotyping assays (Applied Biosystems, Foster City, California).

1.4 BDNF genotyping

For DNA extraction, an aliquot from each saliva sample was added to GeneAll Exgene ™ Clinic SV (Doctor protein INC, Korea) and DNA was extracted according to manufacturer's instructions. Sample DNA extractions were used in subsequent polymerase chain reaction (PCR) to amplify the

target 274-bp BDNF fragment. Following the PCR procedure, the PCR products were run on a 1.5% agarose gel along- side a DNA size ladder at 300 V for 20 min. The agarose gel containing the PCR products was stained with ethidium bromide, visualized under ultraviolet light, and examined for PCR amplification products that represent the amplification of the target 274-bp BDNF fragment. DNA was amplified by using a DNA Engine Tetrad 2 Peltier Thermal Cycler (BIO-RAD, Hercules,CA, USA) and sequenced by using an ABI PRISM 3730XL analyzer (Applied Biosystems, Foster City, CA, USA). Sequencing data were analyzed by using Variant Reporter Software Version 2.1 (Applied Biosystems).

1.5 PET scanners

Each scanner was commissioned by scanning a NEMA phantom and adjusting the reconstruction parameters to obtain a spatial resolution of ~6.5 mm. This optimization was performed before the patients were scanned, and the images received by GE were not subjected to any further post-processing regarding spatial resolution.

1.6 [^{18}F]-flutemetamol PET image acquisition and processing

[^{18}F] FMM was manufactured, and [^{18}F] FMM-PET data were collected and analyzed as described previously [2]. Static PET scans were acquired from 90 to 110 min after 185 MBq of FMM injection. MRI for each participant was used to co-register and define the ROIs and correct partial volume effects that arose from expansion of the cerebrospinal spaces accompanying cerebral atrophy using a geometric transfer matrix.

2 Supplementary tables

2.1 **Supplementary table 1** Number and Distribution of Participants Based on AD Risk Factors

Aβ deposition	Negative (n = 35)	positive (n = 20)	*P* value
APOE ε4 carrier status			
- Non-carrier	23 (65.7%)	6 (30.0%)	0.011
- Carrier	12 (34.3%)	14 (70.0%)	
BDNF polymorphism			
- Met non-carrier	6 (17.1%)	4 (20.0%)	0.792
- Met carrier	29 (82.9%)	16 (80.0%)	

3 References

[1] Byeon H, Jin H, Cho S. Development of Parkinson's disease dementia prediction model based on verbal memory, visuospatial memory, and executive function. Journal of Medical Imaging and Health Informatics 2017;7:1517-21.

[2] Thurfjell L, Lilja J, Lundqvist R, Buckley C, Smith A, Vandenberghe R, et al. Automated quantification of 18F-flutemetamol PET activity for categorizing scans as negative or positive for brain amyloid: concordance with visual image reads. Journal of Nuclear Medicine 2014;55:1623-8.
